# Association of tobacco product use with chronic obstructive pulmonary disease (COPD) prevalence and incidence in Waves 1 through 5 (2013–2019) of the Population Assessment of Tobacco and Health (PATH) Study

**DOI:** 10.1186/s12931-022-02197-1

**Published:** 2022-10-01

**Authors:** Laura M. Paulin, Michael J. Halenar, Kathryn C. Edwards, Kristin Lauten, Cassandra A. Stanton, Kristie Taylor, Dorothy Hatsukami, Andrew Hyland, Todd MacKenzie, Martin C. Mahoney, Ray Niaura, Dennis Trinidad, Carlos Blanco, Wilson M. Compton, Lisa D. Gardner, Heather L. Kimmel, Dana Lauterstein, Daniela Marshall, James D. Sargent

**Affiliations:** 1 Geisel School of Medicine at Dartmouth, The C. Everett Koop Institute at Dartmouth, Hanover, NH USA; 2 Behavioral Health and Health Policy Practice, Westat, Rockville, MD USA; 3 Department of Psychiatry and Behavioral Sciences, University of Minnesota, Minneapolis, MN USA; 4 Department of Health Behavior, Roswell Park Comprehensive Cancer Center, Buffalo, NY USA; 5 New York University School of Global Public Health, New York, NY 10012 USA; 6 University of California at San Diego, La Jolla, CA 92037 USA; 7 National Institute on Drug Abuse, National Institutes of Health, Bethesda, MD USA; 8 Center for Tobacco Products, Food and Drug Administration, Silver Spring, MD USA; 9 Kelly Government Solutions, Rockville, MD USA

**Keywords:** Cigarette, COPD, E-cigarette, Epidemiology, Prevention, Respiratory disease, Smoking-related lung disease, Tobacco

## Abstract

**Background:**

We examined the association of non-cigarette tobacco use on chronic obstructive pulmonary disease (COPD) risk in the Population Assessment of Tobacco and Health (PATH) Study.

**Methods:**

There were 13,752 participants ≥ 40 years with Wave 1 (W1) data for prevalence analyses, including 6945 adults without COPD for incidence analyses; W1–5 (2013–2019) data were analyzed. W1 tobacco use was modeled as 12 mutually-exclusive categories of past 30-day (P30D) single and polyuse, with two reference categories (current exclusive cigarette and never tobacco). Prevalence and incidence ratios of self-reported physician-diagnosed COPD were estimated using weighted multivariable Poisson regression.

**Results:**

W1 mean (SE) age was 58.1(0.1) years; mean cigarette pack-years was similar for all categories involving cigarettes and exclusive use of e-cigarettes (all > 20), greater than exclusive cigar users (< 10); and COPD prevalence was 7.7%. Compared to P30D cigarette use, never tobacco, former tobacco, and cigar use were associated with lower COPD prevalence (RR = 0.33, (95% confidence interval—CI) [0.26, 0.42]; RR = 0.57, CI [0.47, 0.70]; RR = 0.46, CI [0.28, 0.76], respectively); compared to never tobacco use, all categories except cigar and smokeless tobacco use were associated with higher COPD prevalence (RR former = 1.72, CI [1.33, 2.23]; RR cigarette = 3.00, CI [2.37, 3.80]; RR e-cigarette = 2.22, CI [1.44, 3.42]; RR cigarette + e-cigarette = 3.10, CI [2.39, 4.02]; RR polycombusted = 3.37, CI [2.44, 4.65]; RR polycombusted plus noncombusted = 2.75, CI]1.99, 3.81]). COPD incidence from W2-5 was 5.8%. Never and former tobacco users had lower COPD risk compared to current cigarette smokers (RR = 0.52, CI [0.35, 0.77]; RR = 0.47, CI [0.32, 0.70], respectively). Compared to never use, cigarette, smokeless, cigarette plus e-cigarette, and polycombusted tobacco use were associated with higher COPD incidence (RR = 1.92, CI [1.29, 2.86]; RR = 2.08, CI [1.07, 4.03]; RR = 1.99, CI [1.29, 3.07]; RR = 2.59, CI [1.60, 4.21], respectively); exclusive use of e-cigarettes was not (RR = 1.36, CI [0.55, 3.39]).

**Conclusions:**

E-cigarettes and all use categories involving cigarettes were associated with higher COPD prevalence compared to never use, reflecting, in part, the high burden of cigarette exposure in these groups. Cigarette—but not exclusive e-cigarette—use was also strongly associated with higher COPD incidence. Compared to cigarette use, only quitting tobacco was protective against COPD development.

**Supplementary Information:**

The online version contains supplementary material available at 10.1186/s12931-022-02197-1.

## Background

Chronic obstructive pulmonary disease (COPD) is a chronic, progressive respiratory disease associated with inhalational exposure to noxious gases and particles [1]. Cigarette smoking has long been linked to development of COPD [2], but less is known about the impact of other tobacco products, including e-cigarettes, on COPD epidemiology. Increased use of e-cigarette products among smokers who may be seeking to improve health outcomes or quit cigarette smoking has prompted investigation of their relationship with respiratory disease [3]. Given that cigarette smoking is a leading risk factor for COPD development, and that e-cigarette products have been suggested as a lower risk substitute [4, 5], understanding the impact of use of these products on COPD outcomes is a public health priority.

Studies that have examined the relationship between use of e-cigarette products and COPD outcomes show mixed results. A small cohort study of smokers with COPD demonstrated improvement in outcomes among those who switched from cigarette to e-cigarette use [6, 7]. Analyses of large United States (US) surveys suggest that e-cigarette use was associated with greater COPD prevalence among current, former, and never cigarette smokers [8–11]. Specifically, recent analyses of the Population Assessment of Tobacco and Health (PATH) Study found associations between e-cigarette use and COPD [8, 12–14]. While these prior studies have been helpful in identifying trends in tobacco use behavior, many included a large proportion of adults less than 40 years old, an age when COPD is rare [1], and failed to account for important confounding variables including pack-years of cigarette smoking. Similarly, prior work suggesting a relationship between cigar use and COPD has been limited by incomplete assessment of tobacco use history [15]. As such, additional studies examining the impact of tobacco product use over time are needed to clarify these relationships.

COPD is a progressive, incurable disease and a leading cause of morbidity and mortality worldwide that has clearly been associated with cigarette smoking. Understanding how other tobacco products may relate to COPD onset is important for communicating accurate risk information to smokers. The goal of this study, using PATH Study data, is to determine whether commonly used tobacco products, including e-cigarettes, are associated with prevalence and incidence of COPD in US adults aged 40 years and older.

## Methods

### Study design, setting, and participants

The PATH Study is an ongoing, nationally representative, longitudinal cohort study sponsored by the National Institute on Drug Abuse, National Institutes of Health and the Food and Drug Administration’s Center for Tobacco Products. The study collects self-reported information on tobacco-use patterns, health behaviors and medical history. Complete PATH Study design methods have previously been published in detail [16–18]. All adult respondents provided informed consent. The study was conducted by Westat and approved by the Westat Institutional Review Board.

### Primary outcomes

At Wave 1 (W1) of the PATH Study participants were asked: “Has a doctor, nurse or other health professional EVER told you that you had any of the following lung or respiratory conditions? Choose all that apply: COPD, chronic bronchitis, emphysema, asthma, some other lung or respiratory condition, none of the above, don’t know, refused.” At subsequent waves, participants are asked about respiratory disease diagnoses over the past 12 months. For these analyses, COPD, chronic bronchitis, and emphysema diagnoses are combined to create one COPD measure and are referred to as COPD moving forward, similar to prior COPD prevalence studies using National Health and Nutrition Examination Survey (NHANES) data [19, 20], with which our prevalence estimates were compared. COPD at baseline (W1—2013–2014) and new COPD at follow-up (W2—2014–2015; W3—2015–2016; W4—2016–2017; W5—2018–2019) were assessed using a combination of the two questions above. Individuals with history of “some other lung or respiratory condition” were excluded.

**Fig. 1:**
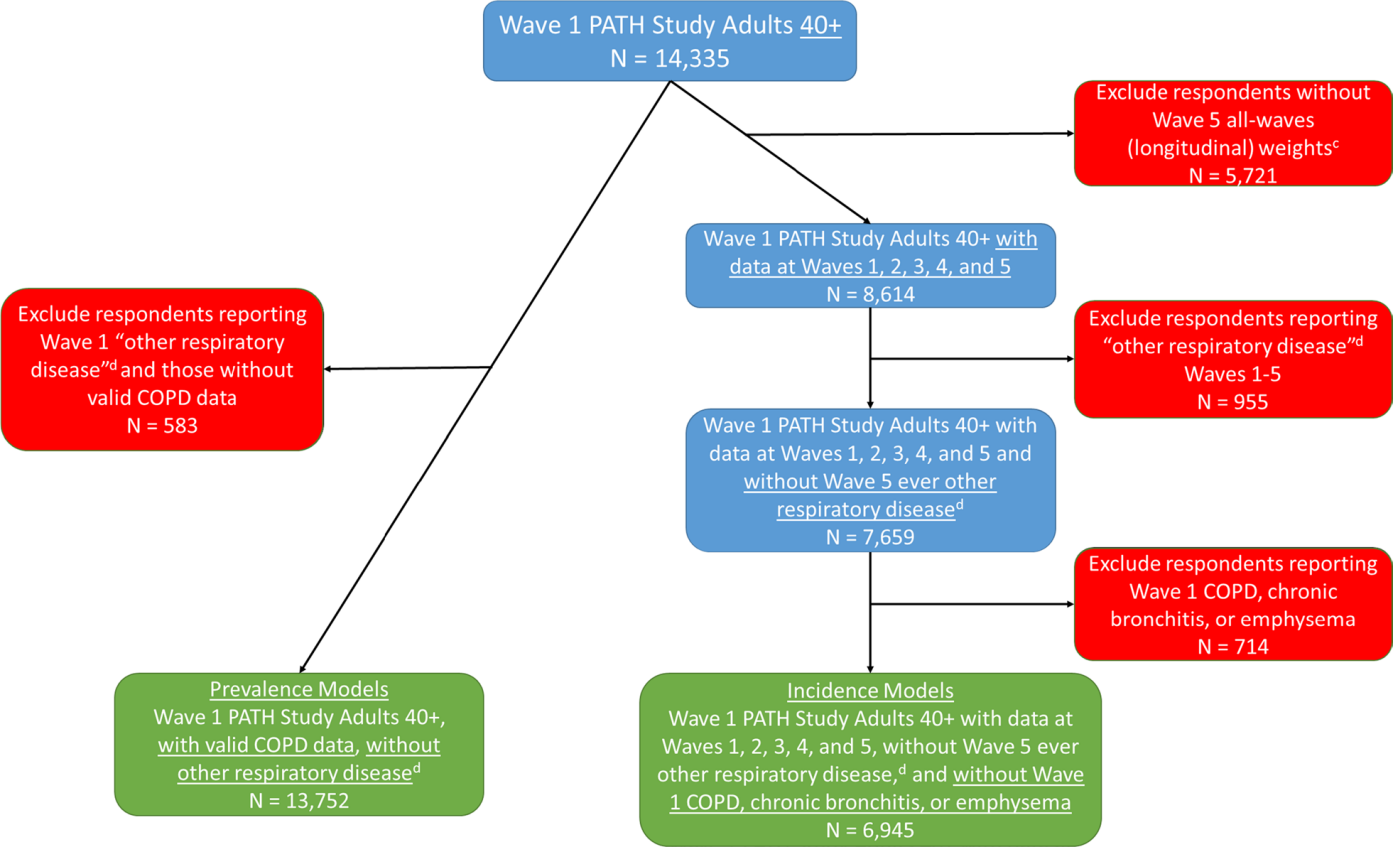
Study flow ^a^This figure illustrates the sample determination from the Population Assessment of Tobacco and Health Study for the prevalence and incidence models (Tables 2 and 3) for COPD ^b^COPD Chronic Obstructive Pulmonary Disease, defined as self-report of emphysema, chronic bronchitis, or COPD; prevalence and incidence models. ^c^Weights adjust for non-response. ^d^Other non-asthma or COPD respiratory diseases

Given that COPD diagnoses are rarely made before age 40, and consistent with national COPD cohorts [20–22], we limited our sample to adults aged 40 years and over at W1. For W1 prevalence estimates, all W1 adults with valid COPD data aged ≥ 40 years were analyzed (N = 13,752); incidence analyses included adults aged ≥ 40 years without a diagnosis of COPD at W1, and for whom data at all five study waves are available (N = 6945) (Fig. 1). Missing data on age, gender, race, Hispanic ethnicity, and adult education were imputed as described in the PATH Study Restricted Use Files User Guide at https://doi.org/10.3886/Series606. The current study uses the W1–5 Adult Restricted Use Files [23].

### Exposures of interest

Participants reported lifetime and past 30-day use of combusted products (cigarettes, traditional cigars, cigarillos, filtered cigars, pipe tobacco, and hookah) and noncombusted products (snus pouches, other smokeless tobacco [loose snus, moist snuff, dip, spit, or chewing tobacco], and e-cigarettes) at W1.

Twelve mutually exclusive categories defined all past and current tobacco use possibilities (never or former experimental [e.g., lifetime use of < 100 cigarettes; or never used other products fairly regularly) users—hereafter referred to as never]; former established [e.g., lifetime use of more than 100 cigarettes; or ever used other products fairly regularly, and who haven’t used any tobacco products in the past 30 days—hereafter referred to as former]; exclusive past 30-day use of products: cigarettes, e-cigarettes, cigars [traditional, cigarillo, and filtered cigars], smokeless [smokeless tobacco and snus], hookah, pipe; past 30-day use of combinations: cigarettes and e-cigarettes; polycombusted; polycombusted and noncombusted; and e-cigarettes and smokeless).

### Covariates

As described in Table 1, covariates were derived from W1 data and include variables that could be associated both with tobacco exposure and COPD. Sociodemographic variables included age, sex, race/ethnicity, education, and urbanicity (urban segments have a minimum population density of at least 2500 people based on US Census blocks and are non-urban otherwise). Other W1 smoke-related exposures included pack-years of cigarette smoking [24], past-month secondhand smoke exposure [25], and marijuana use [26]. Two medical history variables were included: ever asthma diagnosis and a disease comorbidity index score used in prior studies to capture important contributors to morbidity in COPD patients (range 0–9) [27]. For each analysis, models were run with three different levels of adjustment: (1) unadjusted; (2) adjusted for cigarette pack-years; and (3) fully adjusted models adding all other covariates.

**Table 1:** Characteristics of Wave 1 participants with valid data on COPD^a^ status (N = 13,752)

Sociodemographics, Tobacco Use, and Medical History	Adults age 40 and above (N = 13,752)			Adults with self-reported doctor or other health professional diagnosis of COPD^a^ (N = 1506)			Adults without self-reported doctor or other health professional diagnosis of COPD^a^ (N = 12,246)		
	N^b^	Weighted %/Mean	SE	N^b^	Weighted %/Mean	SE	N^b^	Weighted %/Mean	SE
Sociodemographics									
Age in years, mean	13,752	58.1	0.1	1506	63.1	0.5	12,246	57.7	0.1
Sex, N (%) male	6943	47.2%	0.2	627	41.2%	1.7	6316	47.7%	0.3
Race, N (%) non-Hispanic white	9306	71.4%	0.3	1128	79.6%	1.2	8178	70.7%	0.4
Hispanic ethnicity, N (%)	1629	11.4%	0.3	98	6.9%	0.8	1531	11.8%	0.3
Less than high school education, N (%)	2963	17.4%	0.1	492	31.1%	1.8	2471	16.3%	0.2
Live in urban area, N (%)	10,157	75.5%	1.8	1029	69.3%	2.8	9128	76.0%	1.7
Tobacco use									
Ever (100+) cigarette smoker, N (%)	7952	41.1%	0.7	1207	70.2%	1.7	6745	38.7%	0.7
Pack-years^c^, mean	10,861	14.2	0.3	1394	29.4	0.8	9467	12.6	0.3
*Any past 30 day tobacco use categories*									
P30D cigarette use, N (%)	5983	18.3%	0.3	1043	40.5%	1.4	4940	16.5%	0.3
P30D cigar use, N (%)	1265	4.4%	0.1	168	7.1%	0.6	1097	4.2%	0.1
P30D traditional cigar use, N (%)	756	2.6%	0.1	78	3.0%	0.3	678	2.5%	0.1
P30D cigarillo use, N (%)	586	2.0%	0.1	74	3.1%	0.4	512	1.9%	0.1
P30 filtered cigar use, N (%)	403	1.3%	0.1	94	3.9%	0.5	309	1.1%	0.1
P30D hookah use, N (%)	95	0.3%	0.0	14	0.5%	0.1	81	0.3%	0.0
P30D e-cigarette use, N (%)	1393	4.1%	0.1	265	9.8%	0.7	1128	3.7%	0.1
P30D smokeless/snus use, N (%)	746	2.4%	0.1	49	2.1%	0.4	697	2.5%	0.1
*Mutually exclusive P30D tobacco use categories*^d^									
Never use (Never or former experimental tobacco)	3817	51.7%	0.7	120	21.4%	1.9	3697	54.2%	0.7
Former tobacco use	2173	25.2%	0.6	225	33.5%	1.9	1948	24.5%	0.6
Exclusive cigarette use	3773	12.5%	0.3	634	27.4%	1.3	3139	11.3%	0.3
Exclusive e-cigarette use	198	0.6%	0.0	26	1.0%	0.2	172	0.6%	0.0
Exclusive cigar use	391	1.6%	0.1	18	1.0%	0.2	373	1.7%	0.1
Exclusive smokeless/snus use	482	1.7%	0.1	27	1.3%	0.4	455	1.8%	0.1
Exclusive P30D cigarette and e-cigarette use	852	2.7%	0.1	166	6.7%	0.6	686	2.4%	0.1
Polycombusted tobacco use	619	2.0%	0.1	113	4.6%	0.5	506	1.8%	0.1
Polycombusted and noncombusted use	461	1.5%	0.1	70	3.0%	0.3	391	1.3%	0.1
Medical history (self-reported)									
Total COPD^a^ diagnosis, N (%)	1506	7.7%	0.3	1506	100.0%	N/A	N/A	N/A	N/A
COPD^e^ diagnosis, N (%)	939	4.6%	0.2	939	60.1%	1.8	N/A	N/A	N/A
Chronic bronchitis diagnosis, N (%)	762	3.9%	0.2	762	50.6%	1.8	N/A	N/A	N/A
Emphysema diagnosis, N (%)	456	2.0%	0.1	456	26.5%	1.4	N/A	N/A	N/A
BMI, kg/m^2^, mean	13,465	28.4	0.1	1477	29.7	0.3	11,988	28.3	0.1
Comorbidity index,^f^ mean	13,383	1.5	0.0	1466	2.6	0.1	11,917	1.4	0.0

^a^COPD: Chronic Obstructive Pulmonary Disease, defined as self-report of emphysema, chronic bronchitis, or COPD

^b^Unweighted

^c^In this table, mean pack-years is only calculated among ever users of cigarettes

^d^Data are not presented for exclusive hookah, exclusive pipe, and dual e-cigarette + smokeless/snus users due to small sample size. Never tobacco user category includes former experimental (e.g., lifetime use of < 100 cigarettes or never used other products fairly regularly) users; former established user category includes all established users (e.g., lifetime use of more than 100 cigarettes or ever used other products fairly regularly) who did not use any tobacco products in the past 30 days

^e^COPD only, not including emphysema or chronic bronchitis

^f^Comorbidity index (range 0–9) is made up of self-reported ever-diagnosis of coronary heart disease, diabetes, congestive heart failure, stroke, osteoarthritis, hypertension, high cholesterol, stomach ulcers, and Wave 1 obesity based on BMI ≥ 30

### Statistical analysis

We examined the associations between W1 COPD diagnosis and covariates using weighted comparisons of means or proportions, as appropriate. We estimated the weighted prevalence and risk ratio for the association of COPD prevalence and incidence, respectively (hereafter referred to as risk ratio [RR]), with tobacco exposure categories while adjusting for covariates using multivariable Poisson regression. To assess the independent risk of tobacco products and their RR compared to cigarettes, two separate analyses were run, each with a different reference category: (1) never tobacco use and (2) current exclusive cigarette smoking. The prevalence analyses were weighted using the W1 full-sample and replicate weights. The incidence analyses were weighted using the W5 longitudinal (all-waves) full-sample and replicate weights. Standard errors were derived using the Balanced Repeated Replication method [28] with Fay’s adjustment set to 0.3 to increase estimate stability [29]. Pack-years of cigarette smoking (non-zero values) and secondhand smoke exposure variables were Winsorized at the 95th and 99th (100 h) percentiles, respectively, to limit the influence of outliers [27]. Participants who were missing COPD diagnosis data, tobacco product use data, or covariate data were omitted from the analyses. All analyses were conducted using Stata survey data procedures, version 17.0 [30].

### Sensitivity analyses

In a sensitivity analysis, individuals who had contributed to at least one follow-up wave after W1 were included to estimate the association of incident COPD with tobacco exposure categories while adjusting for covariates using Cox’s multivariable proportional hazards model. In this case, the dependent variable is the discrete time indicating the first wave at which COPD was diagnosed and otherwise censored after the last wave they participated in. In addition, separate sensitivity analyses (1) included all adults 18 and older, and (2) excluded adults with baseline (W1) asthma.

## Results

At W1, among adults aged ≥ 40 years with valid COPD data (unweighted N = 13,752), 47.2% were male, 71.4% were white, and 11.4% were of Hispanic ethnicity; 17.4% completed less than high school education, and 75.5% lived in an urban area. Mean body mass index (BMI) was 28.4, and mean comorbidity index score was 1.5. Approximately 41.1% had ever smoked 100 or more cigarettes and among those, there was a mean pack-years of 14.3. In the past 30 days, 18.3% used cigarettes, 4.4% used cigars (the majority of which were traditional), 0.3% used hookah, 4.1% used e-cigarettes, and 2.4% used smokeless tobacco (Table 1).

### Cigarette smoking history among select tobacco use categories

**Fig. 2:**
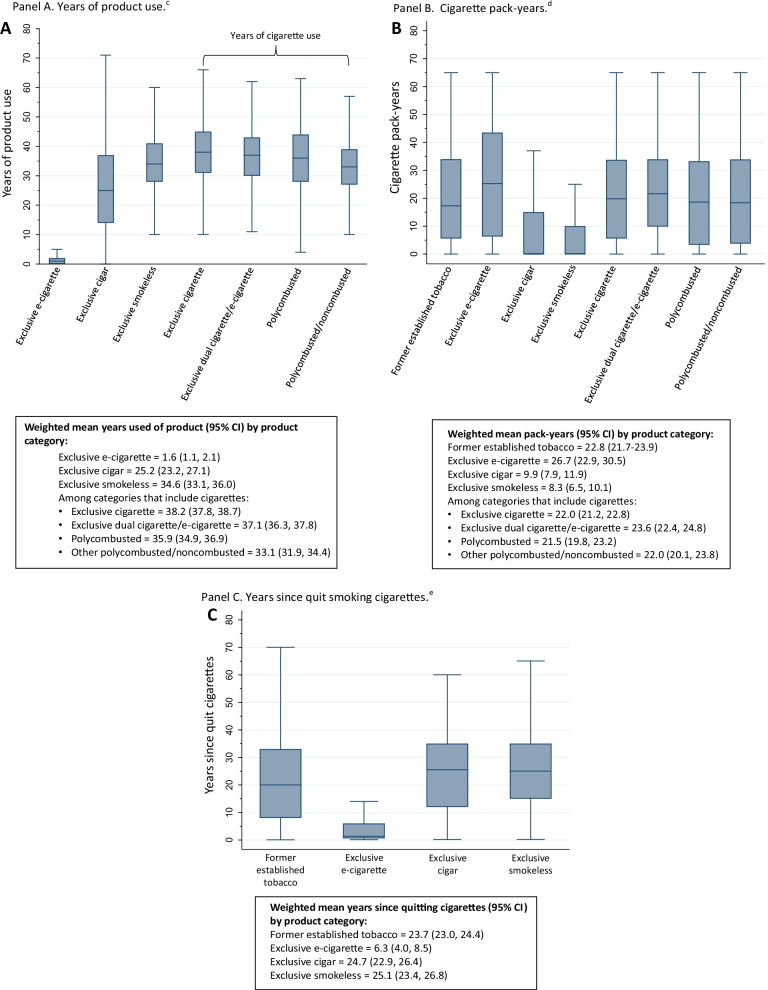
Unweighted distribution for years of product use (**A**), cigarette pack-years (**B**) and years since quit smoking cigarettes (**C**) at Wave 1 for past 30-day exclusive product users. ^a^Outside values are not plotted. **A** Years of product use. ^c^Unweighted Ns: exclusive e-cigarette = 176; exclusive cigar = 352; exclusive smokeless = 475; exclusive cigarette = 3,760; exclusive dual cigarette and e-cigarette = 851; polycombusted = 613; polycombusted and noncombusted = 444. **B** Cigarette pack-years. ^d^Unweighted Ns: former established tobacco = 1920; exclusive e-cigarette = 190; exclusive cigar = 353; exclusive smokeless = 442; exclusive cigarette = 3698; exclusive dual cigarette and e-cigarette = 842; polycombusted = 589; polycombusted and noncombusted = 443. **C** Years since quit smoking cigarettes. ^e^Unweighted Ns: former established tobacco = 2135; exclusive e-cigarette = 190; exclusive cigar = 326; exclusive smokeless = 370.^.b^Former established user category includes all established users (e.g., lifetime use of more than 100 cigarettes or ever used other products fairly regularly) who did not use any tobacco products in the past 30 days

Mean (95% confidence interval—CI) years of product use was greatest for exclusive cigarette users (38.2, CI [37.8, 38.7]) (Fig. 2A). Mean (95% CI) number of pack-years of cigarette smoking was highest in exclusive e-cigarette users (26.7, CI [22.9, 30.5]), dual users of e-cigarettes and cigarettes (23.6, CI [22.4, 24.8]), former tobacco users (22.8, CI [21.7, 23.9]), exclusive cigarette users (22.0, CI [21.2, 22.8]), polycombusted and noncombusted users (22.0, CI [20.1, 23.9]), and polycombusted users (21.5, CI [19.8, 23.2]) (Fig. 2B). Exclusive cigar and smokeless tobacco users had on average > 20 years since quitting cigarettes; exclusive e-cigarette users quit cigarette smoking 6.3 (4.0, 8.5) years ago (Fig. 2C).

### COPD prevalence

W1 COPD prevalence was 7.7%. Of those with self-reported COPD, 60.1%, 50.6%, and 26.5% had COPD, chronic bronchitis, and emphysema, respectively. In general, individuals with COPD were older, predominantly white, and had less than a high school degree. The majority of individuals with COPD were women. Individuals with COPD were also more likely to have ever smoked cigarettes, currently use cigarettes, cigars, hookah and e-cigarettes, and had greater mean pack-years (among ever cigarette smokers) compared to those without COPD. Individuals with COPD were less likely to live in an urban area or use smokeless tobacco in the past 30 days compared to those without COPD. In addition, individuals with COPD had a higher mean BMI and greater comorbidity count (Table 1). PATH Study prevalence of COPD and emphysema diagnoses were very similar to values from NHANES collected during the same time frame. Chronic bronchitis prevalence was lower in the PATH Study as compared to NHANES, making the composite variable of COPD prevalence slightly lower in the PATH Study vs. NHANES (Table 2).

**Table 2:** Comparison of PATH Study COPD^a^ prevalence with NHANES^b^ COPD prevalence^c^

Wave 1 PATH Study 2013–14					NHANES 2013–2014				
Question	PATH Study ID	Response options	Unweighted Ns	Weighted prevalence (SE)	Question	NHANES ID	Response options	Unweighted Ns	Weighted prevalence (SE)
Has a doctor or other health professional ever told you that you had any of the following lung or respiratory conditions? COPD	R01_AX0119_01	Yes No	1076 13,179	5.1% (0.3) 94.9% (0.3)	Has a doctor or other health professional ever told [you/SP] that [you have/s/he/SP has] COPD?	MCQ160o	Yes No	186 3625	5.1% (0.8) 94.9% (0.8)
Has a doctor or other health professional ever told you that you had any of the following lung or respiratory conditions? Chronic bronchitis	R01_AX0119_02	Yes No	876 13,379	4.4% (0.2) 95.7% (0.2)	Has a doctor or other health professional ever told [you/SP] that [you/s/he]...had chronic bronchitis?	MCQ160k	Yes No	249 3560	6.9% (0.5) 93.1% (0.5)
Has a doctor or other health professional ever told you that you had any of the following lung or respiratory conditions? Emphysema	R01_AX0119_03	Yes No	533 13,722	2.4% (0.1) 97.6% (0.1)	Has a doctor or other health professional ever told [you/SP] that [you/s/he]...had emphysema?	MCQ160g	Yes No	90 3722	2.5% (0.5) 97.5% (0.5)
(Derived) Ever been diagnosed with COPD, chronic bronchitis, or emphysema	N/A	Yes No	1699 12,556	8.4% (0.3) 91.6% (0.3)	(Derived) Ever been diagnosed with COPD, chronic bronchitis, or emphysema	N/A	Yes No	383 3424	10.5% (0.9) 89.5% (0.9)

^a^ COPD: Chronic Obstructive Pulmonary Disease

^b^PATH: Population Assessment of Tobacco and Health; NHANES: National Health and Nutrition Examination Survey

^c^Prevalence weighted to reflect estimate for the United States adult population. PATH Study data weighted using Wave 1 cross-sectional weights

ID: Identification, N: number, SE: Standard Error, Y/N: Yes/No, SP: sampled person

PATH Study unweighted Ns and prevalence estimates differ slightly from Table 1 as respondents with a history of “other respiratory disease” were not excluded, so as to mirror NHANES methods

### Association of W1 tobacco product use with COPD prevalence at W1

#### Reference group: past 30-day exclusive cigarette use at W1

When compared to exclusive past 30-day cigarette use, never users (fully adjusted model RR = 0.33, CI [0.26–0.42]), former users (RR = 0.57, CI [0.47, 0.70]), and cigar users (RR = 0.46, CI [0.28, 0.76]) had lower COPD prevalence. Smokeless tobacco use was associated with lower COPD prevalence in the unadjusted and pack-years adjusted models (RR = 0.35, CI [0.18, 0.67] and RR = 0.49, CI [0.26, 0.94], respectively), but not when accounting for all potentially confounding variables (RR = 0.55, CI [0.28, 1.06]). Exclusive past 30-day e-cigarette use, and dual and polyuse categories were not associated with COPD (Table 3; Additional file 1: Table S1**;** all results and effect sizes for individual covariates can be found in online supplement).

**Table 3:** Association between Wave 1 past 30-day tobacco use and COPD^a^ prevalence at Wave 1 of the Population Assessment of Tobacco and Health Study

Twelve mutually exclusive categories of Wave 1 tobacco use^b^	Weighted Percent with COPD (SE)^c^	Covariate adjustment					
		Unadjusted (N = 12,838)		Cigarette pack-years (N = 12,343)		Fully adjusted^d^ (N = 11,822)	
Exclusive cigarette as the reference group		COPD prevalence (RR)	95% CI	COPD prevalence (RR)	95% CI	COPD prevalence (RR)	95% CI
Exclusive P30D cigarette	16.7 (0.7)	Ref	Ref	Ref	Ref	Ref	Ref
Never use (Never or former experimental tobacco)	3.2 (0.3)	0.19***	[0.15,0.24]	0.38***	[0.30,0.48]	0.33***	[0.26,0.42]
Former tobacco use	10.1 (0.7)	0.61***	[0.52,0.71]	0.63***	[0.54,0.74]	0.57***	[0.47,0.70]
Exclusive P30D e-cigarette	12.1 (2.9)	0.73	[0.44,1.20]	0.64	[0.39,1.05]	0.74	[0.46,1.19]
Exclusive P30D cigar	4.6 (1.1)	0.28***	[0.18,0.43]	0.37***	[0.23,0.60]	0.46**	[0.28,0.76]
Exclusive P30D smokeless/snus	5.8 (1.8)	0.35**	[0.18,0.67]	0.49*	[0.26,0.94]	0.55	[0.28,1.06]
Exclusive P30D cigarette and e-cigarette	18.6 (1.7)	1.12	[0.93,1.35]	1.08	[0.90,1.30]	1.03	[0.86,1.24]
P30D polycombusted tobacco use	17.2 (1.7)	1.03	[0.84,1.28]	1.05	[0.86,1.28]	1.12	[0.91,1.38]
P30D polycombusted and noncombusted use	15.6 (1.6)	0.93	[0.76,1.15]	0.92	[0.74,1.15]	0.92	[0.71,1.18]

Never tobacco use as the reference group		COPD prevalence (RR)	95% CI	COPD prevalence (RR)	95% CI	COPD prevalence (RR)	95% CI
Never use (Never or former experimental tobacco)	3.2 (0.3)	Ref	Ref	Ref	Ref	Ref	Ref
Former tobacco use	10.1 (0.7)	3.21***	[2.49,4.14]	1.66***	[1.27,2.17]	1.72***	[1.33,2.23]
Exclusive P30D cigarette	16.7 (0.7)	5.28***	[4.25,6.57]	2.63***	[2.07,3.34]	3.00***	[2.37,3.80]
Exclusive P30D e-cigarette	12.1 (2.9)	3.84***	[2.35,6.28]	1.68*	[1.02,2.77]	2.22***	[1.44,3.42]
Exclusive P30D cigar	4.6 (1.1)	1.46	[0.90,2.37]	0.97	[0.58,1.62]	1.38	[0.82,2.33]
Exclusive P30D smokeless/snus	5.8 (1.8)	1.85	[0.95,3.62]	1.29	[0.67,2.48]	1.63	[0.87,3.07]
Exclusive P30D cigarette and e-cigarette	18.6 (1.7)	5.91***	[4.55,7.67]	2.84***	[2.14,3.76]	3.10***	[2.39,4.02]
P30D polycombusted tobacco use	17.2 (1.7)	5.47***	[4.05,7.37]	2.75***	[2.00,3.77]	3.37***	[2.44,4.65]
P30D polycombusted and noncombusted use	15.6 (1.6)	4.94***	[3.67,6.65]	2.42***	[1.74,3.36]	2.74***	[1.98,3.80]

^a^ COPD: Chronic Obstructive Pulmonary Disease, defined as self-report of emphysema, chronic bronchitis, or COPD

^b^Data are not presented for exclusive hookah, exclusive pipe, and dual e-cigarette + smokeless/snus users due to small sample size. Never tobacco user category includes former experimental (e.g., lifetime use of < 100 cigarettes or never used other products fairly regularly) users; former established user category includes all established users (e.g., lifetime use of more than 100 cigarettes or ever used other products fairly regularly) who did not use any tobacco products in the past 30 days

^c^Overall Wave 1 prevalence was 7.7% (SE = 0.3)

^d^Fully adjusted = Risk ratios (RR) are adjusted for cigarette pack-years (never cigarette users were assigned 0 pack-years), past-week secondhand smoke exposure, past 30-day marijuana use, COPD comorbidity index, ever asthma diagnosis, age, sex, race/ethnicity, education, and urbanicity. See Additional file 1: Tablesecond

S1a and S1b for all covariate estimates

*p < 0.05, ** p < 0.01, *** p < 0.001

#### Reference group: never tobacco use at W1

When compared to never users, all categories featuring cigarette use had higher COPD prevalence in fully adjusted models (former users RR = 1.72, CI [1.33, 2.23]; past 30-day cigarette use RR = 3.00, CI [2.37, 3.80]; dual use of cigarettes and e-cigarettes RR = 3.10, CI [2.39, 4.02]; polycombusted use RR = 3.37, CI [2.44, 4.65]; polycombusted and noncombusted use RR = 2.74, CI [1.98, 3.80]). E-cigarette use was also associated with higher COPD prevalence (RR = 2.22, CI [1.44, 3.42]) (Table 3; Additional file 1: Table S1).

### COPD incidence W2-5

Cumulative COPD incidence in W2-5 was 5.8% (SE = 0.3). Incidence was higher in past 30-day cigarette users vs non-cigarette users (13.6% [SE = 0.6] vs 4.4% [SE = 0.4]), and in past 30-day e-cigarette users vs non-e-cigarette users (12.0% [SE = 1.4] vs 5.6% [SE = 0.3]).

### Association of W1 tobacco product use with COPD incidence at W2–5

#### Reference group: past 30-day exclusive cigarette use at W1

When compared to exclusive cigarette users at W1, never and former users had lower risk of COPD (fully adjusted model RR = 0.52, CI [0.35, 0.79] and RR = 0.47, CI [0.32, 0.70], respectively). In models accounting for pack-years of cigarettes, cigar use was associated with lower COPD risk (RR = 0.29, CI [0.15, 0.59] and RR = 0.42, CI [0.20, 0.88], respectively), but this relationship did not persist following adjustment for all confounders (RR = 0.55, CI [0.24, 1.26]). When unadjusted, past 30-day smokeless tobacco use was associated with lower COPD incidence (RR = 0.55, CI [0.34, 0.90]), but not in fully adjusted models (RR = 1.08, CI [0.58, 2.03]). Past 30-day e-cigarette, and dual and polyusers did not have higher COPD incidence (Table 4; Additional file 1: Table S2).

**Table 4:** Association between Wave 1 past 30-day tobacco use and COPD^a^ incidence Waves 2–5 of the Population Assessment of Tobacco and Health Study

Twelve mutually exclusive categories of Wave 1 tobacco use^b^	Weighted Percent with new onset COPD W2-W5 (SE)^c^	Covariate adjustment					
		Unadjusted (N = 6475)		Cigarette pack-years (N = 6220)		Fully adjusted^d^ (N = 6018)	
Exclusive cigarette as the reference group		COPD incidence (RR)	95% CI	COPD incidence (RR)	95% CI	COPD incidence (RR)	95% CI
Exclusive P30D cigarette	13.4 (0.8)	Ref	Ref	Ref	Ref	Ref	Ref
Never use (Never or former experimental tobacco)	3.8 (0.4)	0.28***	[0.22,0.37]	0.50***	[0.35,0.71]	0.52**	[0.35,0.78]
Former tobacco use	5.5 (0.7)	0.41***	[0.30,0.55]	0.41***	[0.31,0.55]	0.47***	[0.32,0.70]
Exclusive P30D e-cigarette	9.5 (3.5)^†^	0.71	[0.28,1.78]	0.63	[0.26,1.49]	0.71	[0.26,1.92]
Exclusive P30D cigar	3.9 (1.2)^†^	0.29***	[0.15,0.59]	0.42*	[0.20,0.88]	0.55	[0.24,1.26]
Exclusive P30D smokeless/snus	7.4 (1.7)	0.55*	[0.34,0.90]	0.77	[0.46,1.30]	1.08	[0.58,2.03]
Exclusive P30D cigarette and e-cigarette	14.2 (1.9)	1.06	[0.78,1.45]	1.05	[0.78,1.43]	1.04	[0.77,1.40]
P30D polycombusted tobacco use	15.8 (2.4)	1.18	[0.84,1.66]	1.18	[0.84,1.67]	1.35	[0.92,1.99]
P30D polycombusted and noncombusted use	8.5 (1.7)	0.63*	[0.41,0.98]	0.66	[0.43,1.03]	0.77	[0.50,1.19]

Never tobacco use as the reference group		COPD incidence (RR)	95% CI	COPD incidence (RR)	95% CI	COPD incidence (RR)	95% CI
Never use (Never or former experimental tobacco)	3.8 (0.4)	Ref	Ref	Ref	Ref	Ref	Ref
Former tobacco use	5.5 (0.7)	1.44*	[1.04,2.00]	0.83	[0.56,1.22]	0.90	[0.62,1.33]
Exclusive P30D cigarette	13.4 (0.8)	3.53***	[2.73,4.57]	2.02***	[1.41,2.89]	1.92**	[1.29,2.86]
Exclusive P30D e-cigarette	9.5 (3.5)^†^	2.49*	[1.00,6.19]	1.27	[0.54,2.98]	1.36	[0.55,3.39]
Exclusive P30D cigar	3.9 (1.2)^†^	1.03	[0.51,2.10]	0.86	[0.42,1.73]	1.05	[0.49,2.25]
Exclusive P30D smokeless/snus	7.4 (1.7)	1.94*	[1.15,3.26]	1.56	[0.91,2.67]	2.08*	[1.07,4.03]
Exclusive P30D cigarette and e-cigarette	14.2 (1.9)	3.74***	[2.74,5.10]	2.13***	[1.48,3.06]	1.99**	[1.29,3.07]
P30D polycombusted tobacco use	15.8 (2.4)	4.17***	[2.87,6.06]	2.39***	[1.55,3.67]	2.59***	[1.60,4.21]
P30D polycombusted and noncombusted use	8.5 (1.7)	2.24***	[1.45,3.45]	1.34	[0.86,2.08]	1.48	[0.92,2.39]

^a^COPD: Chronic Obstructive Pulmonary Disease, defined as self-report of emphysema, chronic bronchitis, or COPD

^b^Data are not presented for exclusive hookah, exclusive pipe, and dual e-cigarette + smokeless/snus users due to small sample size. Never tobacco user category includes former experimental (e.g., lifetime use of < 100 cigarettes or never used other products fairly regularly) users; former established user category includes all established users (e.g., lifetime use of more than 100 cigarettes or ever used other products fairly regularly) who did not use any tobacco products in the past 30 days

^c^Overall cumulative COPD incidence in W2-5 was 5.8% (SE = 0.3)

^d^Fully adjusted = Risk ratios (RR) are adjusted for cigarette pack-years (never cigarette users were assigned 0 pack-years), past-week secondhand smoke exposure, past 30-day marijuana use, COPD comorbidity index, ever asthma diagnosis, age, sex, race/ethnicity, education, and urbanicity. See Additional file 1: Table S2a and S2b for all covariate estimates

^†^Estimate should be interpreted with caution because it has low statistical precision. It is based on a denominator sample size of less than 50, or the coefficient of variation of the estimate or its complement is larger than 30%

*p < 0.05, ** p < 0.01, *** p < 0.001

#### Reference group: never tobacco use at W1

When compared to never users, most categories featuring cigarette use had higher COPD incidence in fully adjusted models (cigarette use RR = 1.92, CI [1.29, 2.86]; dual cigarette and e-cigarette use RR = 1.99, CI [1.29, 3.07]; polycombusted use RR = 2.59, CI [1.60, 4.21]). Smokeless tobacco use was associated with a higher COPD risk, although this group has a relatively wide CI due to the small number of new COPD cases (RR = 2.08, CI [1.07, 4.03]). There was no relationship between former tobacco, e-cigarette, cigar, or polycombusted and noncombusted use and COPD incidence (Table 4; Additional file 1: Table S2).

### Sensitivity analyses

The incidence of COPD did not meaningfully differ when we included individuals who contributed data to two or more waves (n = 9470) (Additional file 1: Table S3), excluded participants with baseline asthma, and included all adults 18 years and older (data not shown).

## Discussion

In a nationally representative sample of adults aged 40 years and older, past 30-day cigarette use was associated with higher COPD prevalence and incidence compared to never tobacco use. This association remained consistent regardless of what other tobacco products were used in conjunction with cigarettes. These results confirm the expected association between cigarette smoking and COPD outlined in the 1984 Surgeon General’s report and scores of observational studies [2, 31]. COPD prevalence was not found to be lower in exclusive e-cigarette users compared to exclusive cigarette smokers, and when compared to never tobacco use, current e-cigarette use and cigarette and e-cigarette dual use were associated with increased COPD prevalence. Importantly, the COPD prevalence bye-cigarette and cigarette use did not differ; only never use, former use, and cigar use were associated with lower COPD prevalence compared to current cigarette smoking. Similarly, when evaluating the association between tobacco products and COPD incidence, cigarette and cigarette and e-cigarette dual use were associated with greater COPD incidence compared to never tobacco use. Compared to cigarette smoking, only former and never tobacco users had lower COPD incidence; although some categories had relatively small sample sizes, no product was associated with less risk.

E-cigarettes generate particulate matter and trace metals [32–34], which may stimulate inflammation and lung damage to a degree similar to cigarette smoke, potentially contributing to respiratory disease progression [35]. We found an association between e-cigarette use and COPD prevalence, similar to two studies using Behavior Risk Factor Surveillance System (BRFSS) data. Osei et al. found an association between e-cigarette use and higher odds of self-reported COPD in never, former, and current smokers [10]. In a comparable analysis, Xie et al. found an association between e-cigarette use and increased odds of self-reported COPD, including in those who had never smoked cigarettes [11]. Prior work from W1 of the PATH Study also supports our prevalence findings. Perez et al. used propensity-matching to examine the relationship between e-cigarette use and COPD diagnosis, and found that e-cigarette use was associated with higher odds of COPD in both cigarette smokers and non-smokers [8]. Our study adds to this existing knowledge base by comparing COPD prevalence between e-cigarette users and cigarette smokers.

We are uncertain if the observed relationship between e-cigarette use and COPD prevalence is a reflection of the innate risk of e-cigarettes or a marker of higher burden of cigarette exposure. First, COPD develops as a result of decades long exposure to noxious particles and gases [1], such that the duration of e-cigarette use among contemporary users is likely not long enough to have a large influence on COPD onset. Second, the exclusive e-cigarette and exclusive cigarette users in this study had comparable cigarette smoking histories as measured by pack years, and exclusive e-cigarette users had only stopped smoking cigarettes somewhat recently, suggesting that cigarette exposure may be the main driver of the association with COPD. This is similar to the observed association between polyuse and COPD risk, which is likely driven by the cigarette component. While we emphasize the importance of considering pack-years of smoking exposure, we recognize the potential for unmeasured confounding that is not captured by pack-years, which has been shown to be an imperfect measure of overall cigarette smoking history [36].

These uncertainties are highlighted in our findings on COPD incidence. While we found an association between e-cigarette use and COPD incidence when compared to never use in the unadjusted models, this relationship did not persist after careful consideration of additional covariates. These results differ from those of Xie et al., who in their analysis of the first four waves of PATH Study data found an association between e-cigarette use and increased risk of respiratory disease, including COPD [13]. The authors included all adults over the age of 18 in their study, raising uncertainty about the COPD endpoint given that younger age is associated with misdiagnosis of COPD [37]. In addition, while pack-years of cigarette smoking was included as a covariate in the models for current cigarette smokers, it was not for former cigarette smokers. Given that the majority of exclusive e-cigarette users are former smokers, the association observed by Xie et al. may be in part due to a high burden of past cigarette smoking. Indeed, in our study, simply adjusting for cigarette pack-years eliminated the association between exclusive past 30-day e-cigarette smoking and COPD incidence.

Of interest is the association between cigar use and decreased COPD prevalence when compared to cigarettes. In a prior longitudinal study of over 17,000 men who never smoked cigarettes, cigar use was associated with a 45% increased risk of self-reported COPD [38]. Further, in a cross-sectional analysis of Multi-Ethnic Study of Atherosclerosis (MESA) Lung data, Rodriguez et al. found that an increase in cigar-years was associated with airflow obstruction, findings which were attenuated when restricting analysis to never cigarette smokers [15]. In our study, when comparing to never tobacco use, cigar use was not significantly associated with COPD prevalence nor incidence. Most exclusive cigar smokers in the current study had quit cigarette smoking > 20 years ago and had smoked fewer pack-years compared to exclusive cigarette users. In this context, it seems plausible that their risk for COPD tended to be more like former than current cigarette smokers. Furthermore, lower COPD prevalence with exclusive cigar use may be explained by the reduced smoke inhalation and lower frequency of smoking in exclusive users of traditional cigars [39], the predominant cigar type used by the study population [40].

Our study has several strengths. Analysis was limited to adults aged 40 years and older, a timeline consistent with the accepted principles of development of airflow obstruction following years of exposure to noxious particles and gases [1]. Further, our study benefits from the repeated outcome measures afforded by the PATH Study, as well as explicitly accounting for pack-years of smoking and additional confounders not captured by other studies. Using two reference categories (current exclusive cigarette smoking and never tobacco use) highlights the importance of accounting for multiple product use and the unique contributions of each tobacco product to COPD risk.

There are several limitations to note. The PATH Study relies on participant self-report of physician diagnosis and not spirometry to define COPD. While spirometric evidence of airflow obstruction is required to make a COPD diagnosis, spirometry is often underutilized in clinical settings, resulting in both a potential under-diagnosis and over-diagnosis of COPD [19, 41, 42]. As an example, physicians may be more likely to workup or diagnose COPD in a symptomatic individual who smokes compared to a never cigarette smoker, a population that is under-diagnosed with COPD [43] despite estimates that up to 25% of US individuals with COPD have never smoked [44]. The lack of spirometry in the PATH Study along with the potential of diagnostic and recall bias associated with self-report may introduce imprecision in the COPD diagnosis and confound the associations observed in our study. To help mitigate these concerns, the prevalence estimates of COPD in our study are largely consistent with those reported in NHANES analyses, which included spirometry in a subset of participants [19, 20], providing confidence in our study endpoint. Accounting for self-report of asthma as well as multiple other diseases that may share symptoms with COPD in our adjusted analysis helps to address concerns for misclassification of self-report of physician-diagnosed COPD, a potential limitation of survey research in general.

We examined tobacco product use at a single time point (W1), although prior PATH Study research has shown product-specific persistent use over time in adults [45]. Further, our definition of product use also does not allow for detection of a dose-related effect. In addition, residual confounding may exist despite our best efforts to control for cigarette exposure. Knowing that respiratory symptoms may influence an individual’s ability and choice to inhale tobacco products, the potential remains for reverse causality. The analysis considered the e-cigarette devices and flavorings used in W1 and these may have changed over time in ways relevant to COPD development. Further, the sample size for several tobacco products was somewhat limited. Exclusive e-cigarette use was relatively uncommon in adults ≥ 40 years of age, which limits the power to determine if they are a lower harm product in population studies. Future studies should extend this analysis with more waves of data to capture both the association of a longer of e-cigarette use with COPD risk as well to account for new adopters of e-cigarettes in a dynamic tobacco industry.

## Conclusions

In summary, our longitudinal analysis of PATH Study W1-5 data supports existing, fundamental knowledge about the relationship between cigarette smoking, alone or in combination with other tobacco products, and COPD prevalence and incidence. These results further the need for continued focus on prevention of tobacco product initiation as well as effective behavioral and pharmacologic tobacco cessation therapies to decrease COPD incidence. We found an association between exclusive e-cigarette use and COPD prevalence when compared to never tobacco use, which may be due to a high burden of pack-years among users of these products; notably, we did not find an association between e-cigarette use and COPD incidence. Future work that examines a longer duration of e-cigarette use would be of value to better characterize the impact of these products on COPD outcomes.

## Supplementary Information

**Additional file 1.** Association of tobacco product use with chronic obstructive pulmonary disease (COPD) prevalence and incidence in Waves 1 through 5 (2013–2019) of the Population Assessment of Tobacco and Health (PATH) Study.

## Data Availability

Details on accessing the PATH Study data are described in the PATH Study Restricted Use Files website located at https://doi.org/10.3886/ICPSR36231.v29. Access to these data is restricted. Users interested in obtaining these data must complete a Restricted Data Use Agreement.

## Abbreviations

BMI: Body mass index
BRFSS: Behavior Risk Factor Surveillance System
CI: Confidence interval
COPD: Chronic obstructive pulmonary disease
MESA: Multi-Ethnic Study of Atherosclerosis
NHANES: National Health and Nutrition Examination Survey
P30D: Past 30-day
PATH: Population Assessment of Tobacco and Health
RR: Risk ratio
W1–5: Waves 1–5
