# Correction to: Association of tobacco product use with chronic obstructive pulmonary disease (COPD) prevalence and incidence in waves 1 through 5 (2013–2019) of the population assessment of tobacco and health (PATH) study

**DOI:** 10.1186/s12931-025-03166-0

**Published:** 2025-03-08

**Authors:** Laura M. Paulin, Michael J. Halenar, Kathryn C. Edwards, Kristin Lauten, Cassandra A. Stanton, Kristie Taylor, Dorothy Hatsukami, Andrew Hyland, Todd MacKenzie, Martin C. Mahoney, Ray Niaura, Dennis Trinidad, Carlos Blanco, Wilson M. Compton, Lisa D. Gardner, Heather L. Kimmel, Dana Lauterstein, Daniela Marshall, James D. Sargent

**Affiliations:** 1 Geisel School of Medicine at Dartmouth, The C. Everett Koop Institute at Dartmouth, Hanover, NH USA; 2 Behavioral Health and Health Policy Practice, Westat, Rockville, MD USA; 3 Department of Psychiatry and Behavioral Sciences, University of Minnesota, Minneapolis, MN USA; 4 Department of Health Behavior, Roswell Park Comprehensive Cancer Center, Buffalo, NY USA; 5 New York University School of Global Public Health, New York, NY 10012 USA; 6 University of California at San Diego, La Jolla, CA 92037 USA; 7 National Institute on Drug Abuse, National Institutes of Health, Bethesda, MD USA; 8 Center for Tobacco Products, Food and Drug Administration, Silver Spring, MD USA; 9 Kelly Government Solutions, Rockville, MD USA

**Correction to: Respiratory Research (2022) 23:273**

10.1186/s12931-022-02197-1

In the original publication of this article [1], the co-author “Ray Niaura” has provided the following declaration statement “Between mid-2015 and 2020, Dr. Niaura frequently communicated with Juul Labs personnel, for which there was no compensation, and received hospitality in the form of meals at some meetings” to be added under “Competing interests” section. The updated completing interests is given below.

**Competing interests**

Martin C. Mahoney has provided expert testimony on the health effects of smoking in lawsuits filed against the tobacco industry. He has also received research support from Pfizer, Inc., for a clinical trial of smoking cessation, and has previously served on external advisory panels sponsored by Pfizer to promote smoking cessation in clinical settings. Raymond Niaura receives funding from the Food and Drug Administration Center for Tobacco Products via contractual mechanisms with Westat and the National Institutes of Health. Within the past 3 years, he has served as a paid consultant to the Government of Canada via a contract with Industrial Economics Inc. and has received an honorarium for a virtual meeting from Pfizer Inc. Dr. Niaura was an unpaid grant reviewer for the Foundation for a Smoke Free World. Wilson Compton reports long-term stock holdings in General Electric, 3 M Company, and Pfizer Incorporated, unrelated to this article. Between mid-2015 and 2020, Dr. Niaura frequently communicated with Juul Labs personnel, for which there was no compensation, and received hospitality in the form of meals at some meetings.
