# Supplementary material for: Association of tobacco product use with chronic obstructive pulmonary disease (COPD) prevalence and incidence in Waves 1 through 5 (2013–2019) of the Population Assessment of Tobacco and Health (PATH) Study
Source: Respir Res. 2022 Oct 1;23:273. doi: 10.1186/s12931-022-02197-1 (PMC9526897; doi:10.1186/s12931-022-02197-1)
Supplement: Supplementary file 1 — Additional file 1. Association of tobacco product use with chronic obstructive pulmonary disease (COPD) prevalence and incidence in Waves 1 through 5 (2013–2019) of the Population Assessment of Tobacco and Health (PATH) Study. [file 12931_2022_2197_MOESM1_ESM.docx]

**Table S1**: Association between Wave 1 past 30-day tobacco use and COPD^a^ prevalence at Wave 1 of the Population Assessment of Tobacco and Health Study

|  |  | Covariate adjustment | | | | | |
| --- | --- | --- | --- | --- | --- | --- | --- |
| **Twelve mutually exclusive categories of Wave 1 tobacco use^b^** | Weighted Percent with COPD (SE)^c^ | Unadjusted  (N=12838) | | Cigarette pack-years  (N=12343) | | Fully adjusted^d^  (N=11822) | |
| **Exclusive cigarette as the reference group** |  | COPD prevalence (RR) | 95% CI | COPD prevalence (RR) | 95% CI | COPD prevalence (RR) | 95% CI |
| Exclusive P30D cigarette | 16.7 (0.7) | Ref | Ref | Ref | Ref | Ref | Ref |
| Never use (Never or former experimental tobacco) | 3.2 (0.3) | 0.19*** | [0.15,0.24] | 0.38*** | [0.30,0.48] | 0.33*** | [0.26,0.42] |
| Former tobacco use | 10.1 (0.7) | 0.61*** | [0.52,0.71] | 0.63*** | [0.54,0.74] | 0.57*** | [0.47,0.70] |
| Exclusive P30D e-cigarette | 12.1 (2.9) | 0.73 | [0.44,1.20] | 0.64 | [0.39,1.05] | 0.74 | [0.46,1.19] |
| Exclusive P30D cigar | 4.6 (1.1) | 0.28*** | [0.18,0.43] | 0.37*** | [0.23,0.60] | 0.46** | [0.28,0.76] |
| Exclusive P30D smokeless/snus | 5.8 (1.8) | 0.35** | [0.18,0.67] | 0.49* | [0.26,0.94] | 0.55 | [0.28,1.06] |
| Exclusive P30D cigarette and e-cigarette | 18.6 (1.7) | 1.12 | [0.93,1.35] | 1.08 | [0.90,1.30] | 1.03 | [0.86,1.24] |
| P30D polycombusted tobacco use | 17.2 (1.7) | 1.03 | [0.84,1.28] | 1.05 | [0.86,1.28] | 1.12 | [0.91,1.38] |
| P30D polycombusted and noncombusted use | 15.6 (1.6) | 0.93 | [0.76,1.15] | 0.92 | [0.74,1.15] | 0.92 | [0.71,1.18] |
| **Never tobacco use as the reference group** |  | COPD prevalence (RR) | 95% CI | COPD prevalence (RR) | 95% CI | COPD prevalence (RR) | 95% CI |
| Never use (Never or former experimental tobacco) | 3.2 (0.3) | Ref | Ref | Ref | Ref | Ref | Ref |
| Former tobacco use | 10.1 (0.7) | 3.21*** | [2.49,4.14] | 1.66*** | [1.27,2.17] | 1.72*** | [1.33,2.23] |
| Exclusive P30D cigarette | 16.7 (0.7) | 5.28*** | [4.25,6.57] | 2.63*** | [2.07,3.34] | 3.00*** | [2.37,3.80] |
| Exclusive P30D e-cigarette | 12.1 (2.9) | 3.84*** | [2.35,6.28] | 1.68* | [1.02,2.77] | 2.22*** | [1.44,3.42] |
| Exclusive P30D cigar | 4.6 (1.1) | 1.46 | [0.90,2.37] | 0.97 | [0.58,1.62] | 1.38 | [0.82,2.33] |
| Exclusive P30D smokeless/snus | 5.8 (1.8) | 1.85 | [0.95,3.62] | 1.29 | [0.67,2.48] | 1.63 | [0.87,3.07] |
| Exclusive P30D cigarette and e-cigarette | 18.6 (1.7) | 5.91*** | [4.55,7.67] | 2.84*** | [2.14,3.76] | 3.10*** | [2.39,4.02] |
| P30D polycombusted tobacco use | 17.2 (1.7) | 5.47*** | [4.05,7.37] | 2.75*** | [2.00,3.77] | 3.37*** | [2.44,4.65] |
| P30D polycombusted and noncombusted use | 15.6 (1.6) | 4.94*** | [3.67,6.65] | 2.42*** | [1.74,3.36] | 2.74*** | [1.98,3.80] |
| **Other Smoke Exposure** |  |  |  |  |  |  |  |
| Cigarette pack-years (per 5 years) | N/A | - | - | 1.14*** | [1.12,1.16] | 1.08*** | [1.07,1.10] |
| Past week secondhand smoke exposure (per 5 hours) | N/A | - | - | - | - | 1.02*** | [1.01,1.04] |
| Past 30-day marijuana use^e^ |  |  |  |  |  |  |  |
| No | 7.4 (0.3) | - | - | - | - | Ref | Ref |
| Yes | 12.7 (1.3) | - | - | - | - | 1.25* | [1.03,1.53] |
| **Medical History** |  |  |  |  |  |  |  |
| COPD comorbidity index^f^ | N/A | - | - | - | - | 1.30*** | [1.25,1.35] |
| Ever Asthma Diagnosis |  |  |  |  |  |  |  |
| No | 6.0 (0.3) | - | - | - | - | Ref | Ref |
| Yes | 22.3 (1.2) | - | - | - | - | 3.06*** | [2.65,3.53] |
| **Sociodemographics** |  |  |  |  |  |  |  |
| Age (in decades) |  |  |  |  |  |  |  |
| 40-49 | 3.3 (0.3) | - | - | - | - | Ref | Ref |
| 50-59 | 7.3 (0.5) | - | - | - | - | 1.80*** | [1.48,2.19] |
| 60-69 | 9.7 (0.6) | - | - | - | - | 2.17*** | [1.76,2.67] |
| 70-79 | 12.9 (1.1) | - | - | - | - | 2.85*** | [2.23,3.66] |
| 80+ | 11.4 (1.8) | - | - | - | - | 3.34*** | [2.42,4.62] |
| Sex |  |  |  |  |  |  |  |
| Female | 8.5 (0.4) | - | - | - | - | Ref | Ref |
| Male | 6.6 (0.4) | - | - | - | - | 0.70*** | [0.61,0.81] |
| Race/Ethnicity |  |  |  |  |  |  |  |
| Non-Hispanic White | 8.5 (0.4) | - | - | - | - | Ref | Ref |
| Non-Hispanic Black | 6.9 (0.6) | - | - | - | - | 0.73*** | [0.61,0.87] |
| Non-Hispanic Other | 4.4 (0.6) | - | - | - | - | 0.69* | [0.52,0.92] |
| Hispanic | 4.5 (0.5) | - | - | - | - | 0.72* | [0.56,0.94] |
| Education |  |  |  |  |  |  |  |
| Less than high school | 13.3 (0.9) | - | - | - | - | 2.60*** | [1.96, 3.45] |
| High school graduate | 9.5 (0.6) | - | - | - | - | 2.21*** | [1.67, 2.92] |
| Some college | 7.8 (0.5) | - | - | - | - | 1.98*** | [1.46, 2.67] |
| Bachelor’s degree or more | 2.5 (0.3) | - | - | - | - | Ref | Ref |
| Urbanicity |  |  |  |  |  |  |  |
| Urban | 7.0 (0.3) | - | - | - | - | Ref | Ref |
| Not Urban | 9.6 (0.6) | - | - | - | - | 0.99 | [0.84,1.18] |

^a^ COPD= Chronic Obstructive Pulmonary Disease, defined as self-report of emphysema, chronic bronchitis, or COPD

^b^ Data are not presented for exclusive hookah, exclusive pipe, and dual e-cigarette + smokeless/snus users due to small sample size. Never tobacco user category includes former experimental (e.g., lifetime use of < 100 cigarettes or never used other products fairly regularly) users; former established user category includes all established users (e.g., lifetime use of more than 100 cigarettes or ever used other products fairly regularly) who did not use any tobacco products in the past 30 days.

^c^ Overall Wave 1 prevalence was 7.7% (SE=0.3).

^d^ Fully adjusted = Risk ratios (RR) are adjusted for all of the variables in the table.

^e^ Marijuana use variable does not distinguish between combusted and noncombusted use.

^f^ For this table, COPD comorbidity index values of 7, 8, or 9 were set to 6 because of low Ns.

* p<0.05, ** p<0.01, *** p<0.001

**Table S2**: Association between Wave 1 past 30-day tobacco use and COPD^a^ incidence Waves 2-5 of the Population Assessment of Tobacco and Health Study

|  |  | Covariate adjustment | | | | | |
| --- | --- | --- | --- | --- | --- | --- | --- |
| **Twelve mutually exclusive categories of Wave 1 tobacco use^b^** | Weighted Percent with new onset COPD W2-W5 (SE)^c^ | Unadjusted  (N=6475) | | Cigarette pack-years  (N=6220) | | Fully adjusted^d^  (N=6018) | |
| **Exclusive cigarette as the reference group** |  | COPD incidence (RR) | 95% CI | COPD incidence (RR) | 95% CI | COPD incidence (RR) | 95% CI |
| Exclusive P30D cigarette | 13.4 (0.8) | Ref | Ref | Ref | Ref | Ref | Ref |
| Never use (Never or former experimental tobacco) | 3.8 (0.4) | 0.28*** | [0.22,0.37] | 0.50*** | [0.35,0.71] | 0.52** | [0.35,0.78] |
| Former tobacco use | 5.5 (0.7) | 0.41*** | [0.30,0.55] | 0.41*** | [0.31,0.55] | 0.47*** | [0.32,0.70] |
| Exclusive P30D e-cigarette | 9.5 (3.5)^†^ | 0.71 | [0.28,1.78] | 0.63 | [0.26,1.49] | 0.71 | [0.26,1.92] |
| Exclusive P30D cigar | 3.9 (1.2)^†^ | 0.29*** | [0.15,0.59] | 0.42* | [0.20,0.88] | 0.55 | [0.24,1.26] |
| Exclusive P30D smokeless/snus | 7.4 (1.7) | 0.55* | [0.34,0.90] | 0.77 | [0.46,1.30] | 1.08 | [0.58,2.03] |
| Exclusive P30D cigarette and e-cigarette | 14.2 (1.9) | 1.06 | [0.78,1.45] | 1.05 | [0.78,1.43] | 1.04 | [0.77,1.40] |
| P30D polycombusted tobacco use | 15.8 (2.4) | 1.18 | [0.84,1.66] | 1.18 | [0.84,1.67] | 1.35 | [0.92,1.99] |
| P30D polycombusted and noncombusted use | 8.5 (1.7) | 0.63* | [0.41,0.98] | 0.66 | [0.43,1.03] | 0.77 | [0.50,1.19] |
| **Never tobacco use as the reference group** |  | COPD incidence (RR) | 95% CI | COPD incidence (RR) | 95% CI | COPD incidence (RR) | 95% CI |
| Never use (Never or former experimental tobacco) | 3.8 (0.4) | Ref | Ref | Ref | Ref | Ref | Ref |
| Former tobacco use | 5.5 (0.7) | 1.44* | [1.04,2.00] | 0.83 | [0.56,1.22] | 0.90 | [0.62,1.33] |
| Exclusive P30D cigarette | 13.4 (0.8) | 3.53*** | [2.73,4.57] | 2.02*** | [1.41,2.89] | 1.92** | [1.29,2.86] |
| Exclusive P30D e-cigarette | 9.5 (3.5)^†^ | 2.49* | [1.00,6.19] | 1.27 | [0.54,2.98] | 1.36 | [0.55,3.39] |
| Exclusive P30D cigar | 3.9 (1.2)^†^ | 1.03 | [0.51,2.10] | 0.86 | [0.42,1.73] | 1.05 | [0.49,2.25] |
| Exclusive P30D smokeless/snus | 7.4 (1.7) | 1.94* | [1.15,3.26] | 1.56 | [0.91,2.67] | 2.08* | [1.07,4.03] |
| Exclusive P30D cigarette and e-cigarette | 14.2 (1.9) | 3.74*** | [2.74,5.10] | 2.13*** | [1.48,3.06] | 1.99** | [1.29,3.07] |
| P30D polycombusted tobacco use | 15.8 (2.4) | 4.17*** | [2.87,6.06] | 2.39*** | [1.55,3.67] | 2.59*** | [1.60,4.21] |
| P30D polycombusted and noncombusted use | 8.5 (1.7) | 2.24*** | [1.45,3.45] | 1.34 | [0.86,2.08] | 1.48 | [0.92,2.39] |
| **Other Smoke Exposure** |  |  |  |  |  |  |  |
| Cigarette pack-years (per 5 years) | N/A | - | - | 1.13*** | [1.09,1.17] | 1.10*** | [1.05,1.14] |
| Past week secondhand smoke exposure (per 5 hours) | N/A | - | - | - | - | 1.05** | [1.02,1.09] |
| Past 30-day marijuana use^e^ |  |  |  |  |  |  |  |
| No | 5.8 (0.3) | - | - | - | - | Ref | Ref |
| Yes | 8.8 (1.7) | - | - | - | - | 0.94 | [0.59,1.52] |
| **Medical History** |  |  |  |  |  |  |  |
| COPD comorbidity index^f^ | N/A | - | - | - | - | 1.13* | [1.02,1.25] |
| Ever Asthma Diagnosis |  |  |  |  |  |  |  |
| No | 5.2 (0.3) | - | - | - | - | Ref | Ref |
| Yes | 13.4 (1.7) | - | - | - | - | 2.35*** | [1.72,3.21] |
| **Sociodemographics** |  |  |  |  |  |  |  |
| Age (in decades) |  |  |  |  |  |  |  |
| 40-49 | 4.1 (0.4) | - | - | - | - | Ref | Ref |
| 50-59 | 6.2 (0.6) | - | - | - | - | 1.41* | [1.05,1.91] |
| 60-69 | 6.0 (0.6) | - | - | - | - | 1.50* | [1.10,2.03] |
| 70-79 | 9.0 (1.3) | - | - | - | - | 2.50*** | [1.63,3.84] |
| 80+ | 7.9 (3.1)^†^ | - | - | - | - | 2.00 | [0.81,4.91] |
| Sex |  |  |  |  |  |  |  |
| Female | 7.3 (0.4) | - | - | - | - | Ref | Ref |
| Male | 4.4 (0.4) | - | - | - | - | 0.53*** | [0.42,0.66] |
| Race/Ethnicity |  |  |  |  |  |  |  |
| Non-Hispanic White | 5.9 (0.4) | - | - | - | - | Ref | Ref |
| Non-Hispanic Black | 7.9 (0.9) | - | - | - | - | 1.17 | [0.85,1.61] |
| Non-Hispanic Other | 4.7 (1.3) | - | - | - | - | 1.02 | [0.54,1.93] |
| Hispanic | 4.6 (0.8) | - | - | - | - | 0.80 | [0.52,1.22] |
| Education |  |  |  |  |  |  |  |
| Less than high school | 13.2 (1.2) | - | - | - | - | 2.62*** | [1.77, 3.88] |
| High school graduate | 5.9 (0.7) | - | - | - | - | 1.20 | [0.80, 1.81] |
| Some college | 5.3 (0.6) | - | - | - | - | 1.24 | [0.85, 1.80] |
| Bachelor’s degree or more | 3.0 (0.4) | - | - | - | - | Ref | Ref |
| Urbanicity |  |  |  |  |  |  |  |
| Urban | 5.7 (0.4) | - | - | - | - | Ref | Ref |
| Not Urban | 6.4 (0.9) | - | - | - | - | 0.91 | [0.66,1.27] |

^a^ COPD= Chronic Obstructive Pulmonary Disease, defined as self-report of emphysema, chronic bronchitis, or COPD

^b^ Data are not presented for exclusive hookah, exclusive pipe, and dual e-cigarette + smokeless/snus users due to small sample size. Never tobacco user category includes former experimental (e.g., lifetime use of < 100 cigarettes or never used other products fairly regularly) users; former established user category includes all established users (e.g., lifetime use of more than 100 cigarettes or ever used other products fairly regularly) who did not use any tobacco products in the past 30 days.

^c^ Overall cumulative COPD incidence in W2-5 was 5.8% (SE=0.3).

^d^ Fully adjusted = Risk ratios (RR) are adjusted for all of the variables in the table.

^e^ Marijuana use variable does not distinguish between combusted and noncombusted use.

^f^ For this table, COPD comorbidity index values of 7, 8, or 9 were set to 6 because of low Ns.

† Estimate should be interpreted with caution because it has low statistical precision. It is based on a denominator sample size of less than 50, or the coefficient of variation of the estimate or its complement is larger than 30%.

* p<0.05, ** p<0.01, *** p<0.001

**Table S3:** Sensitivity analyses comparing the Wave 1 past 30-day mutually exclusive tobacco use exposure and COPD^a^ incidence Waves 2-5 of the PATH Study presented in Tables 3 and 4 of the manuscript and Cox hazard ratio models including those contributing data from two or more waves.

|  | Covariate adjustment | | | | | | | | | | | |
| --- | --- | --- | --- | --- | --- | --- | --- | --- | --- | --- | --- | --- |
|  | Unadjusted | | | | Cigarette pack-years | | | | Fully Adjusted^b^ | | | |
|  | Poisson  (N=6475) | | Cox  (N=9470) | | Poisson  (N=6220) | | Cox  (N=9086) | | Poisson  (N=6018) | | Cox  (N=8762) | |
| **Twelve mutually exclusive categories of Wave 1 tobacco use^c^** | COPD incidence (RR) | 95% CI | COPD incidence (HR) | 95% CI | COPD incidence (RR) | 95% CI | COPD incidence (HR) | 95% CI | COPD incidence (RR) | 95% CI | COPD incidence (HR) | 95% CI |
| **Exclusive cigarette as the reference group** |  |  |  |  |  |  |  |  |  |  |  |  |
| Exclusive P30D cigarette | Ref | Ref | Ref | Ref | Ref | Ref | Ref | Ref | Ref | Ref | Ref | Ref |
| Never use (Never or former experimental tobacco) | 0.28*** | [0.22,0.37] | 0.30*** | [0.24,0.38] | 0.49*** | [0.34,0.70] | 0.52*** | [0.40,0.68] | 0.52** | [0.35,0.77] | 0.52*** | [0.38,0.70] |
| Former tobacco use | 0.41*** | [0.30,0.55] | 0.44*** | [0.34,0.56] | 0.41*** | [0.31,0.55] | 0.44*** | [0.34,0.58] | 0.47*** | [0.32,0.70] | 0.46*** | [0.34,0.63] |
| Exclusive P30D e-cigarette | 0.71 | [0.28,1.78] | 0.88 | [0.50,1.55] | 0.62 | [0.26,1.47] | 0.81 | [0.46,1.42] | 0.70 | [0.26,1.90] | 0.80 | [0.42,1.55] |
| Exclusive P30D cigar | 0.29*** | [0.15,0.59] | 0.24*** | [0.14,0.43] | 0.42* | [0.20,0.87] | 0.30*** | [0.17,0.55] | 0.55 | [0.24,1.26] | 0.40** | [0.21,0.76] |
| Exclusive P30D smokeless/snus | 0.55* | [0.34,0.90] | 0.56** | [0.38,0.82] | 0.77 | [0.46,1.30] | 0.79 | [0.53,1.18] | 1.08 | [0.58,2.03] | 1.04 | [0.67,1.61] |
| Exclusive P30D cigarette and e-cigarette | 1.06 | [0.78,1.45] | 1.12 | [0.88,1.43] | 1.06 | [0.78,1.44] | 1.11 | [0.87,1.42] | 1.04 | [0.77,1.41] | 1.07 | [0.82,1.39] |
| P30D polycombusted tobacco use | 1.18 | [0.84,1.66] | 1.19 | [0.91,1.56] | 1.19 | [0.84,1.68] | 1.21 | [0.92,1.59] | 1.36 | [0.92,2.00] | 1.33 | [0.99,1.79] |
| P30D polycombusted and noncombusted use | 0.63* | [0.41,0.98] | 0.70 | [0.48,1.02] | 0.66 | [0.43,1.02] | 0.70 | [0.48,1.02] | 0.77 | [0.50,1.18] | 0.79 | [0.53,1.17] |
| **Never tobacco use as the reference group** | COPD incidence (RR) | 95% CI | COPD incidence (HR) | 95% CI | COPD incidence (RR) | 95% CI | COPD incidence (Hazard) | 95% CI | COPD incidence (RR) | 95% CI | COPD incidence (HR) | 95% CI |
| Never use (Never or former experimental tobacco) | Ref | Ref | Ref | Ref | Ref | Ref | Ref | Ref | Ref | Ref | Ref | Ref |
| Former tobacco use | 1.44* | [1.04,2.00] | 1.45* | [1.07,1.95] | 0.84 | [0.57,1.24] | 0.85 | [0.61,1.18] | 0.91 | [0.62,1.33] | 0.89 | [0.64,1.25] |
| Exclusive P30D cigarette | 3.53*** | [2.73,4.57] | 3.31*** | [2.65,4.13] | 2.05*** | [1.43,2.92] | 1.92*** | [1.47,2.51] | 1.93** | [1.30,2.88] | 1.93*** | [1.43,2.60] |
| Exclusive P30D e-cigarette | 2.49* | [1.00,6.19] | 2.91*** | [1.62,5.23] | 1.27 | [0.54,3.00] | 1.55 | [0.84,2.86] | 1.36 | [0.55,3.39] | 1.55 | [0.77,3.11] |
| Exclusive P30D cigar | 1.03 | [0.51,2.10] | 0.81 | [0.45,1.44] | 0.86 | [0.43,1.75] | 0.58 | [0.32,1.07] | 1.05 | [0.49,2.26] | 0.77 | [0.41,1.46] |
| Exclusive P30D smokeless/snus | 1.94* | [1.15,3.26] | 1.84** | [1.22,2.79] | 1.58 | [0.92,2.69] | 1.52 | [0.99,2.33] | 2.09* | [1.08,4.04] | 2.01** | [1.28,3.17] |
| Exclusive P30D cigarette and e-cigarette | 3.74*** | [2.74,5.10] | 3.72*** | [2.78,4.96] | 2.17*** | [1.52,3.09] | 2.13*** | [1.54,2.94] | 2.01** | [1.31,3.09] | 2.06*** | [1.43,2.97] |
| P30D polycombusted tobacco use | 4.17*** | [2.87,6.06] | 3.95*** | [2.90,5.39] | 2.43*** | [1.59,3.73] | 2.32*** | [1.63,3.29] | 2.62*** | [1.62,4.25] | 2.57*** | [1.74,3.80] |
| P30D polycombusted and noncombusted use | 2.24*** | [1.45,3.45] | 2.31*** | [1.54,3.47] | 1.35 | [0.87,2.09] | 1.34 | [0.87,2.07] | 1.48 | [0.91,2.39] | 1.52 | [0.94,2.45] |
| **Other Smoke Exposure** |  |  |  |  |  |  |  |  |  |  |  |  |
| Cigarette pack-years (per 5 years) | - | - | - | - | 1.12*** | [1.08,1.16] | 1.12*** | [1.09,1.15] | 1.09*** | [1.05,1.13] | 1.10*** | [1.07,1.14] |
| Past week secondhand smoke exposure (per 5 hours) | - | - | - | - | - | - | - | - | 1.05** | [1.02,1.09] | 1.04*** | [1.02,1.07] |
| Past 30-day marijuana use^d^ |  |  |  |  |  |  |  |  |  |  |  |  |
| No | - | - | - | - | - | - | - | - | Ref | Ref | Ref | Ref |
| Yes | - | - | - | - | - | - | - | - | 0.94 | [0.59,1.52] | 0.94 | [0.69,1.28] |
| **Medical History** |  |  |  |  |  |  |  |  |  |  |  |  |
| COPD comorbidity index^e^ | - | - | - | - | - | - | - | - | 1.13* | [1.02,1.24] | 1.15*** | [1.07,1.24] |
| Ever Asthma Diagnosis |  |  |  |  |  |  |  |  |  |  |  |  |
| No | - | - | - | - | - | - | - | - | Ref | Ref | Ref | Ref |
| Yes | - | - | - | - | - | - | - | - | 2.35*** | [1.72,3.21] | 2.98*** | [2.28,3.89] |
| **Sociodemographics** |  |  |  |  |  |  |  |  |  |  |  |  |
| Age (in decades) |  |  |  |  |  |  |  |  |  |  |  |  |
| 40-49 | - | - | - | - | - | - | - | - | Ref | Ref | Ref | Ref |
| 50-59 | - | - | - | - | - | - | - | - | 1.41* | [1.05,1.91] | 1.35* | [1.05,1.73] |
| 60-69 | - | - | - | - | - | - | - | - | 1.49* | [1.10,2.02] | 1.50** | [1.14,1.98] |
| 70-79 | - | - | - | - | - | - | - | - | 2.49*** | [1.62,3.83] | 2.17*** | [1.50,3.15] |
| 80+ | - | - | - | - | - | - | - | - | 1.99 | [0.81,4.88] | 1.53 | [0.86,2.74] |
| Sex |  |  |  |  |  |  |  |  |  |  |  |  |
| Female | - | - | - | - | - | - | - | - | Ref | Ref | Ref | Ref |
| Male | - | - | - | - | - | - | - | - | 0.53*** | [0.42,0.66] | 0.53*** | [0.44,0.65] |
| Race/Ethnicity |  |  |  |  |  |  |  |  |  |  |  |  |
| Non-Hispanic White | - | - | - | - | - | - | - | - | Ref | Ref | Ref | Ref |
| Non-Hispanic Black | - | - | - | - | - | - | - | - | 1.17 | [0.84,1.61] | 1.13 | [0.87,1.47] |
| Non-Hispanic Other | - | - | - | - | - | - | - | - | 1.02 | [0.54,1.93] | 1.06 | [0.66,1.72] |
| Hispanic | - | - | - | - | - | - | - | - | 0.79 | [0.52,1.21] | 0.97 | [0.68,1.38] |
| Education |  |  |  |  |  |  |  |  |  |  |  |  |
| Less than high school | - | - | - | - | - | - | - | - | 2.62*** | [1.77,3.88] | 2.18*** | [1.54,3.08] |
| High school graduate | - | - | - | - | - | - | - | - | 1.20 | [0.80,1.81] | 1.38 | [0.99,1.91] |
| Some college | - | - | - | - | - | - | - | - | 1.24 | [0.85,1.80] | 1.28 | [0.95,1.74] |
| Bachelor’s degree or more | - | - | - | - | - | - | - | - | Ref | Ref | Ref | Ref |
| Urbanicity |  |  |  |  |  |  |  |  |  |  |  |  |
| Urban | - | - | - | - | - | - | - | - | Ref | Ref | Ref | Ref |
| Not Urban | - | - | - | - | - | - | - | - | 0.91 | [0.66,1.27] | 1.01 | [0.81,1.26] |

^a^ COPD= Chronic Obstructive Pulmonary Disease, defined as self-report of emphysema, chronic bronchitis, or COPD

^b^ Fully adjusted = Risk ratios (RR) and Hazard ratios (HR) are adjusted for all of the variables in the table.

^c^ Data are not presented for exclusive hookah, exclusive pipe, and dual e-cigarette + smokeless/snus users due to small sample size. Never tobacco user category includes former experimental (e.g., lifetime use of < 100 cigarettes or never used other products fairly regularly) users; former established user category includes all established users (e.g., lifetime use of more than 100 cigarettes or ever used other products fairly regularly) who did not use any tobacco products in the past 30 days.

^d^ Marijuana use variable does not distinguish between combusted and noncombusted use.

^e^ For this table, COPD comorbidity index values of 7, 8, or 9 were set to 6 because of low Ns.

* p<0.05, ** p<0.01, *** p<0.001
